# Functions of METTL1/WDR4 and QKI as m7G modification - related enzymes in digestive diseases

**DOI:** 10.3389/fphar.2024.1491763

**Published:** 2025-01-09

**Authors:** Wenyan Zhou, Yan Yi, Wenyu Cao, Xiaolin Zhong, Ling Chen

**Affiliations:** ^1^ Department of Metabolism and Endocrinology, The First Affiliated Hospital, Hengyang Medical School, University of South China, Hengyang, China; ^2^ Institute Center of Clinical Medicine, The First Affiliated Hospital, Hengyang Medical School, University of South China, Hengyang, China; ^3^ Clinical Anatomy and Reproductive Medicine Application Institute, Hengyang Medical School, University of South China, Hengyang, China

**Keywords:** m7G, METTL1, WDR4, QKI, cancer, digestive diseases

## Abstract

N^7^-methylguanosine (m7G) modification is one of the most prevalent forms of chemical modification in RNA molecules, which plays an important role in biological processes such as RNA stability, translation regulation and ribosome recognition. Methyl-transferation of m7G modification is catalyzed by the enzyme complex of methyltransferase-like 1 (METTL1) and WD repeat domain 4 (WDR4), and Quaking (QKI) recognizes internal m7G methylated mRNA and regulates mRNA translation and stabilization. Recent studies have found that m7G modification - related enzymes are associated with the onset and progression of digestive cancer, such as colorectal cancer, liver cancer, and other digestive diseases such as ulcerative colitis. This review will focus on the latest research progress on the roles of m7G methyltransferase METTL1/WDR4 and recognized enzyme QKI in digestive diseases.

## 1 Introduction

Epigenetic modification of ribonucleic acid (RNA) has been widely recognized as a key mechanism regulating gene expression and cell function. Among them, m7G modification is a common and important form of modification, especially at the 5′cap end of mRNA. This modification is catalyzed by m7G methyltransferase and is widely present in various RNA molecules, including messenger RNA (mRNA), transfer RNA (tRNA), ribosomal RNA (rRNA) and microRNA (miRNA).

In recent years, researchers have conducted in-depth studies on the role of m7G modification in the regulation of cell biological processes. m7G modification is not only involved in the stability and transport of RNA, but also closely related to transcription, translation and subcellular localization of RNA. Wherein, the m7G methylated proteins METTL1 and WDR4 play a key role in this regulatory network. Despite the initial understanding of the mechanisms underlying m7G modifications and related functional proteins, their specific role in the pathogenesis of digestive diseases remains a challenging area. Recent studies have shown that m7G modification plays a key regulatory role in the regulation of digestive diseases. In-depth research on the regulatory mechanisms of m7G modification and its functional proteins in the occurrence and development of digestive diseases is needed. Therefore, the purpose of this review is to summarize the role of m7G-modified functional enzymes in digestive diseases and explore their potential therapeutic applications. Through systemic analysis of the molecular mechanism of m7G modification in the occurrence of digestive system diseases, we are expected to reveal new therapeutic targets and provide new ideas for the treatment and prevention of related diseases.

## 2 Detection method of m7G modification

The latest research methods for detecting m7G modifications primarily consist of two major categories: antibody-based analysis and chemical-based detection. The former encompasses techniques such as m7G-MeRIP-Seq and m7G-miCLIPseq (Saglam and Akgul, 2024), which cleverly utilize specific antibodies to capture and enrich RNA fragments containing m7G modifications, followed by high-throughput sequencing technology to precisely map the distribution of m7G on RNA molecules. Recent studies have shown that MBL and Biovision antibodies exhibit high efficacy in detecting m7G marks in specific RNA species, making global detection and relative quantitation of m7G across different biological samples both simple and rapid (D'Ambrosi et al., 2024). On the other hand, chemical detection approaches, including m7G-MAP-seq (Enroth et al., 2019), m7G-quant-seq (Zhang et al., 2022), and AlkAniline-Seq (Marchand et al., 2021), rely on chemical reduction, reverse transcription, or molecular cleavage techniques to strive for precise identification of m7G modification sites at the single-nucleotide level (Lin et al., 2019). However, a challenge faced by these techniques is their relatively high misidentification rate for non-target modifications. Fortunately, as an emerging technology, borohydride sequencing (Bo-Seq) has been proven to effectively circumvent the issue of variable mutation rates, thereby ensuring accurate identification of m7G sites (D'Ambrosi et al., 2024). Additionally, the rise of nanopore sequencing technology and fluorescence resonance energy transfer (FRET) has opened up new horizons for the dynamic monitoring of m7G modifications (Wang W. et al., 2021). These technologies offer the possibility of real-time tracking of changes in m7G modifications, further enriching our understanding of this important RNA modification process.

## 3 METTL1/WDR4 as writer in m7G modification

### 3.1 Structure of METTL1/WDR4

METTL1 is a protein composed of 1,292 nucleotides and 276 amino acids. The METTL1 gene is located on human chromosome 12q13. The gene has seven exons and six introns, and its sequence is similar to the yeast open reading frame (ORF) YDL201w. METTL1, like other members of the Class I methyltransferase (MTase) family, has a highly conserved Rossmann-like fold consisting of seven β-sheet and six α-helices (Bahr et al., 1999; Li R. et al., 2023; Qi et al., 2023). WDR4 is a protein composed of 412 amino acids, which contains seven WD40 domains inside. The WDR4 gene is located on the human chromosome 21q22.3 (Michaud et al., 2000). WDR4 consists of seven blades (B1-B7) of a β-propeller structure and four seven β-sheet, in which the B2-B5 region is adjacent to METTL1 and is highly stable (Hu et al., 2017; Jin et al., 2023; Li Z. et al., 2023). The interaction between R170 and E167 of WDR4 with the K143 domain of METTL1 enhances the enzymatic activity of the METTL1/WDR4 complex (Ruiz-Arroyo et al., 2023). The METTL1/WDR4 complex is highly similar to the yeast tRNA m7G methyltransferase complex Trm8-Trm82 (Campeanu et al., 2021; Jin et al., 2023).

### 3.2 Mechanism of METTL1/WDR4

**FIGURE 1:**
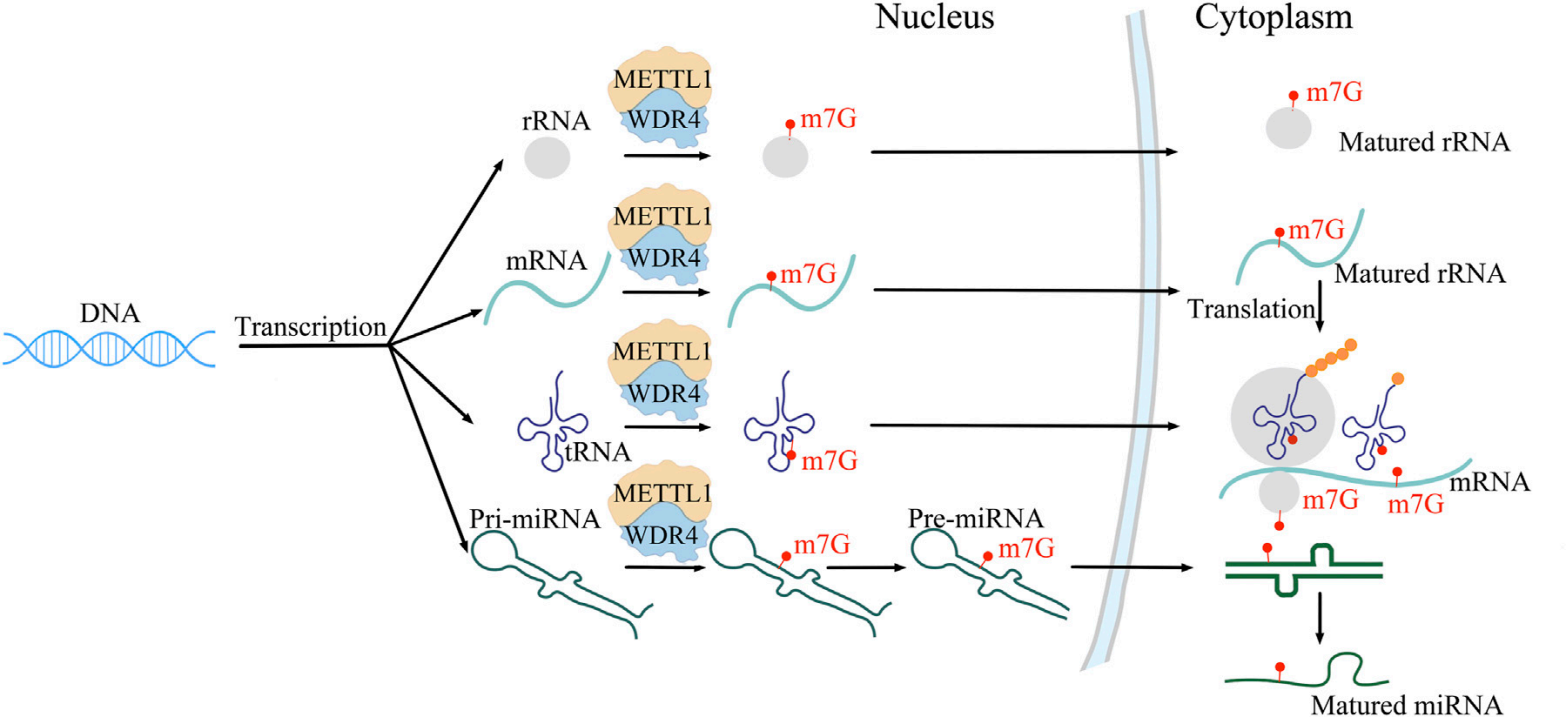
Localization sites of and functions m7G modification in mRNA, tRNA, rRNA and miRNA.

METTL1 and WDR4, as m7G writer enzymes, play a crucial role in cell regulation. METTL1 is a methyltransferase that introduces methyl groups to the N7 position of RNA molecules to form the m7G structure. This modification not only plays an important role in the stability and translation of RNA, but also plays an important role in the regulation of gene expression levels (Pandolfini et al., 2019; Zhang et al., 2019; Orellana et al., 2021; Gao et al., 2022). WDR4 is one of the cofactors of METTL1. WDR4 enhances the binding force of METTL1 and SAM (S-adenosylmethionine), thereby promoting the methyltransferase activity of the METTL1/WDR4 complex on tRNA m7G. (Campeanu et al., 2021; Jin et al., 2023). WDR4 is essential for normal embryonic development. The mutations of WDR4 can cause microcephalic primordial dwarfism. The METTL1/WDR4 complex catalyzes m7G methylation of mRNA, tRNA, rRNA, and miRNA (Li J. et al., 2023; Weng et al., 2023) (Figure 1). METTL1 influences the translation process through methylation of mRNA, tRNA, and miRNA (Lin et al., 2018; Pandolfini et al., 2019; Du et al., 2023; Ruiz-Arroyo et al., 2023), and it also plays a crucial role in the maturation of miRNA and the maintenance of its secondary structure. In the process of transcription initiation, m7G methyltransferase catalyzes the formation of mRNA 5′-terminal methylation cap, which can prevent mRNA from being destroyed by nuclease, and regulate the splicing and translation of messenger ribonucleic acid precursors. mRNA with hat structure is more easily recognized by the initiation factor of protein synthesis, thus promoting protein synthesis. M7G is present not only at the 5′end of mRNA, but also inside the mRNA. The METTL1/WDR4 complex is responsible for adding m7G to the inner region of mRNA, promoting mRNA maturation and translation. The METTL1/WDR4 complex performs m7G modification at position 46 of the tRNA ring (Cartlidge et al., 2005) to enhance mRNA translation by weakening ribosome suspension (Wang et al., 2023a; Zhang M. et al., 2023), thereby regulating self-renewal and differentiation of embryonic stem cells and playing a role in promoting tumor progression. The METTL1/WDR4 complex mediates m7G labeling at position 1,639 of 18S rRNA, promotes maturation of 18S rRNA and 40S ribosomal subunits, and enhances mRNA translation. In addition, METTL1/WDR4 complex can also perform m7G methylation modification on miRNA, promote miRNA maturation and maintain its secondary structure. METTL1 plays a role in the growth of acute myeloid leukemia cells and in the self-renewal and differentiation of embryonic stem cells. The METTL1/WDR4 complex has the ability to methylate mRNA and miRNA *in vitro*, and can directly participate in the modification of mRNA and miRNA (Boulias and Greer, 2019). A study has shown that the mitochondrial inner membrane protein TMEM11 can interact with METTL1, increasing m7G modification (Chen et al., 2023). In addition, the enzyme activity of METTL1 is inhibited by phosphorylation of AKT, suggesting that its enzyme activity may be regulated by signaling pathways (Boulias and Greer, 2019; Pandolfini et al., 2019; Cheng et al., 2022). Recent literature research confirms that METTL1 enzyme activity is not only regulated by mTOR signaling (Garcia-Vilchez et al., 2023a), but also modulated by the P300/SP1 complex (Zhang R. et al., 2023). During the cell cycle, METTL1 protects specific mRNA from decaying through tRNA methylation. METTL1 regulates the N7-methylation of tRNA, affecting the survival of cancer cells under stress. It has been found that m7G in tRNA can protect them from stress-induced cutting and processing of 5′tRNA fragments. The lack of m7G methylation of tRNA activates the stress response pathway, making cancer cells more susceptible to stress. In addition, loss of METTL1 inhibits tumor growth, cell viability, and increases cytotoxic stress in the body. This study reveals the importance of m7G methylation of tRNA in the stress response and suggests that targeting METTL1 may increase cancer sensitivity to chemotherapy (Garcia-Vilchez et al., 2023b). In addition, the METTL1/WDR4 complex influences tRNA function, ribosome suspension, and mRNA translation in mouse embryonic stem cells, and is particularly sensitive to the translation of cell cycle genes and genes associated with brain abnormalities (Lin et al., 2018; Chen Y. et al., 2022). In addition, the upregulated expression of METTL1/WDR4 complex in a variety of cancers is associated with malignancy and poor survival (Gao et al., 2022). Loss of METTL1 and WDR4 impairs cancer cell growth, tumorgenesis, and malignant transformation (Katsara and Schneider, 2021; Ma et al., 2021; Orellana et al., 2021). Therefore, the METTL1/WDR4 complex may be a potential target for cancer treatment (D'Ambrosi et al., 2024).

## 4 QKI as reader in m7G modification

### 4.1 Structure of QKI

QKI, also known as Quaking, is a member of the signal transduction and activation of RNA (STAR) signal transduction activation family, and also belongs to the heterogeneous nuclear ribonucleoprotein K homology (KH) domain protein family. There are three main alternative splicing isomers, QKI-5, QKI-6 and QKI-7, with different carboxy-terminal domains. QKI-5, QKI-6 and QKI-7 are composed of 956, 785 and 542 amino acids, respectively with molecular weights of approximately 55kDa, 48 kDa and 32kDa, respectively. QKI-5 is mainly in the nucleus, while QKI-6/7 is present in both the nucleus and cytoplasm, mainly in the cytoplasm (Cox et al., 1999; Saccomanno et al., 1999; Galarneau and Richard, 2005).

### 4.2 Mechanism of QKI

QKI is an RNA-binding protein, whose structure includes RNA recognition regions and multiple RNA-binding protein subunits, endowing it with the ability to regulate RNA splicing, stability, and translation within cells (Chen and Richard, 1998; de Bruin et al., 2016). Its unique molecular conformation enables it to play a key role in biological processes such as nervous system development (Ebersole et al., 1996), cell cycle, and stem cell differentiation (Darbelli and Richard, 2016; Shingu et al., 2017). QKI has specific recognition function in m7G modified internal mRNA. It binds to m7G-modified mRNA rich in “GA” sequences, which in turn regulates the metabolism of the target subset. QKI-6 and QKI-7 interact with stress particle protein G3BP1 to promote transport of internal m7G-modified transcripts into stress particles, which leads to increased stability and decreased translation of mRNA under doxorubicin-induced stress. Specifically, QKI-7 inhibits the translation efficiency of GSK3B and TEAD1 genes essential to the Hippo signaling pathway, increasing the sensitivity of cancer cells to chemotherapy drugs such as doxorubicin and sorafenib (Zhao et al., 2023). Therefore, QKI regulates mRNA metabolism and influences tumor cell resistance to chemotherapy. In addition, QKI delays macrophage differentiation through a negative feedback mechanism to CCAAT/enhancer binding protein (C/EBP) α. QKI-5 can decrease Keap1 mRNA translation and promote the activation of Nrf2, thus leading to the decrease of reactive oxygen species (ROS) level. QKI-5 enhances antioxidant capacity by regulating Nrf2-Keap one pathway (Zhang M. et al., 2023). There are three downregulated genes (NUDT7, NUDT12 and POLR2H) and two upregulated genes (QKI and PRKCB) in m7G-related immune differentially expressed genes (DEG). QKI regulates the polarization of macrophages through the nuclear factor κB (NF-κB) pathway and influences the severity of inflammation, and further inhibits the expression and phosphorylation of p65 and effectively inhibits the NF-kB pathway, thereby inhibiting inflammation (Wang W. et al., 2021; Yang and Yuan, 2023).

## 5 The role of m7G in digestive cancers

### 5.1 Hepatocellular carcinoma

The incidence of hepatocellular carcinoma (HCC) ranks sixth worldwide, making it one of the most commonly diagnosed cancers and the third highest cancer mortality rate (Sung et al., 2021). In HCC, upregulation of METTL1 and WDR4 is associated with advanced tumor stage, vascular invasion status, and poor patient survival (Wang H. et al., 2023; Wang H. et al., 2024; Wang W. et al., 2024). The silencing of METTL1 and WDR4 can inhibit the proliferation, migration and invasion of HCC cells.

METTL1 knockdown reduces m7G modification of tRNA, affects m7G modified tRNA expression in HCC cells, and leads to tRNA m7G modification promoting the translation of target mRNA with high frequency of m7G-related codons (Chen et al., 2021; Chen J. et al., 2022; Luo et al., 2022; Zhang et al., 2024). METTL1 plays a role in HCC in a variety of ways, including regulating the cell cycle, promoting tumorigenesis, enhancing invasion and migration, and influencing chemoresistance. First, in terms of promoting tumorigenesis, METTL1 knockdown reduces downstream signaling activity by reducing mRNA translation in the EGF/EGFR and VEGFA/VEGFR1 signaling pathways, leading to inhibition of HCC proliferation and metastasis. In addition, METTL1 also exhibits carcinogenic activity on the PTEN/AKT signaling pathway. In terms of regulating the cell cycle, METTL1 knockdown inhibits Cyclin A2 (CCNA2) translation, leading to cell cycle arrest (Tian et al., 2019; Luo et al., 2022; Yang et al., 2022). In terms of enhancing invasion and migration, METTL1-mediated m7G tRNA modifications selectively modulate the translation of key genes in the epithelial to mesenchymal transition (EMT) process in a codon frequency dependent manner, and targeting the METTL1-m7G-SLUG/SNAIL axis to prevent HCC metastasis after radiofrequency thermal ablation therapy provides a molecular basis (Zhu et al., 2023). Finally, in terms of affecting chemoresistance, METTL1 can promotes the translation of genes in the EGFR pathway, promoting HCC resistance to Lenvatinib (Huang et al., 2023). After radiation therapy, METTL1 promotes DNA double-strand break (DSB) repair, which promotes non-homologous end junctions of DNA by increasing mRNA translation of DNA-dependent protein kinase catalytic subunits (DNA-PKCs) and DNA ligase IV, leading to radiation resistance in HCC (Liao et al., 2023). Recent studies suggest that blocking the axis of METTL1-TGF-β2-PMN-MDSC may be a therapeutic strategy to restore anti-tumor immunity and reduce HCC recurrence after radio frequency ablation (RFA) therapy (Li R. et al., 2023; Zeng et al., 2023).

WDR4 is also highly expressed in HCC and increases the methylation level of m7G. WDR4 has two main effects on HCC, promoting the proliferation of tumor cells and their chemoresistance. First, in terms of promoting tumor cell proliferation, WDR4 induces G1/G0 cell cycle transformation, inhibits apoptosis, promotes proliferation of HCC cells, and significantly increases tumor size and weight. In addition, WDR4 activates the transcription of tumor promoting gene MYC, and promotes the binding of EIF2A and Cyclin B1 (CCNB1) mRNA to enhance the translation of CCNB1, thus promoting the development of HCC. By promoting p53 ubiquitination, CCNB1 increases the phosphorylation levels of PI3K and AKT in HCC and decreases the expression of p53 protein, thus promoting the proliferation of tumor cells (Xia et al., 2021; Li et al., 2022a, Luo et al., 2022). Second, in terms of chemoresistance, WDR4 can increase the translation of TRIM28, thereby enhancing the stemness of tumor stem cells, promoting HCC resistance to Lenvatinib and tumor progression (Dong et al., 2024). WDR4 may also promote resistance to sorafenib in HCC by enhancing CCNB1 translation (Xia et al., 2021).

### 5.2 Intrahepatic cholangiocarcinoma

Intrahepatic cholangiocarcinoma (ICC) is the second most common type of primary liver cancer with a fatal malignancy (El-Diwany et al., 2019). The effect of m7G on ICC is mainly reflected by the tumor promoting function of METTL1 and WDR4, mainly affecting cell cycle arrest and translation damage, with cell cycle arrest occurring after translation damage. They enhance the growth, migration, and invasion of ICC cells by regulating frequency-dependent, specific oncogenic mRNA translation of m7G modified tRNA codons. This process reduces ribosome pauses at the m7G modified tRNA decoding codon, affects the expression of m7G modifications and specific tRNA, and thus regulates the translation of carcinogenic mRNA (Goodarzi et al., 2016). In addition, the expression levels of cell cycle genes such as CCNA2, Cyclin D2 (CCND2),Cyclin-dependent kinase 6 (CDK6) and Cyclin-dependent kinase 8 (CDK8), as well as the phosphorylation levels of epidermal growth factor receptor (EGFR) signaling pathway genes and the translation levels of their downstream targets such as AKT and mTOR, were significantly downregulated (Dai S. et al., 2021). Some studies have found that translation damage is due to the accumulation of ribosomes in the 80S, and ribosome collision during elongation is related to increased phosphorylation of eIF2a and impaired translation initiation (Wu et al., 2020; Yan and Zaher, 2021). These findings provide new targets and research directions for future ICC therapy, suggesting that METTL1/WDR4-mediated m7G tRNA modification plays an important role in the progression of ICC (Luo et al., 2022). Knockout of METTL1 in ICC cells significantly increased the proportion of G2/M phase cells, and also increased the apoptosis of ICCs cells. In addition, METTL1 deletion can reduce the level of m7G modification in its target tRNA, resulting in reduced tRNA levels and affecting the physiological function and progression of ICC (Dai Z. et al., 2021). In the face of the grim prognosis of ICC, it is important to focus on early diagnosis, the development of more effective adjuvant therapy, and the search for novel drugs to improve patient survival (Mavros et al., 2014; Rizvi et al., 2018).

### 5.3 Colorectal cancer

Colorectal cancer (CC) is the third most common cancer worldwide, but the second leading cause of cancer death, after lung cancer (Sung et al., 2021). In CC, m7G-related genes, METTL1 and WDR4 are closely related to the occurrence and development of the disease, mainly in promoting tumorigenesis, enhancing the ability of invasion and migration, and producing chemoresistance. First, in terms of promoting tumorigenesis, METTL1 promotes colorectal cancer cell proliferation and G1/S translation by inhibiting checkpoint kinase 2 (CHEK2) expression (Jiang et al., 2024). In addition, m7G-related genes, such as MYC, XPO1 and PIK3CA, are closely related to the proliferation and prognosis of CC, and studies have shown that these genes play an important role in disease progression (Li and Wang, 2022). Second, in terms of affecting chemoresistance, overexpressed METTL1 increased the chemical sensitivity of cisplatin resistant CC cells by regulating the miR-149-3p/S100A4/p53 signaling pathway (Liu et al., 2019). Finally, in terms of enhancing the ability of invasion and migration, the METTL1/WDR4 complex promotes the action of let-7e miRNA in an m7G-dependent manner and inhibits the proliferation, migration, and invasion of CC cells by negatively regulating HMGA2. Therefore, the METTL1/let-7e miRNA/HMGA2 axis is closely related to the development of CC (Liu et al., 2020). In addition, long non-coding RNA (lncRNA) associated with m7G are also closely associated with CC, and their main role is involved in the regulation of cellular transcription and translation. The researchers constructed a 21-feature lncRNA risk model, which can effectively assess the prognosis of CC patients, and the application of this model is expected to have a positive impact on the diagnosis and treatment of CC in the future (Liu et al., 2022). These research results provide a new theoretical and practical basis for the prognosis assessment, diagnosis and treatment of CC, and will have a positive impact on the prevention and treatment of this disease.

### 5.4 Pancreatic cancer

Pancreatic cancer (PC) is an insidious and rapidly developing malignant tumor, which is often difficult to detect in time because there are no obvious symptoms in the early stage (Khan et al., 2021). The expression of m7G-associated lncRNA in PC cells was evaluated, and SNHG8 was found to inhibit the biological function of PC cells. So, SNHG8 has a gene suppressor effect in PC (Lu et al., 2023). In addition, high levels of WBSCR22 and TRMT112 expression led to downregulation of ISG15, significantly reducing malignant expression in PC. This finding suggests that the WBSCR22/TRMT112/ISG15 axis holds promise as an innovative strategy for the future treatment of PC (Khan et al., 2022).

### 5.5 Esophageal squamous cell carcinoma

Esophageal squamous cell carcinoma (ESCC) is a serious health threat worldwide, accounting for 90% of all esophageal cancer cases worldwide (Smyth et al., 2017). In ESCC tissues, studies have shown that the expression of tRNA m7G methyltransferase complex protein METTL1/WDR4 is significantly increased. These two proteins promote the development of ESCC by promoting tRNA m7G methyltransferase activity (Han et al., 2022). METTL1/WDR4 mainly promotes tumorigenesis and regulates apoptosis of ESCC. On the one hand, when METTL1 or WDR4 is knocked down, it results in reduced expression of m7G modified tRNA, thereby reducing translation of the oncogenic subset of transcripts enriched in the RPTOR/ULK1/autophagy pathway, thereby promoting tumorigenesis (Han et al., 2022, Luo et al., 2022). On the other hand, the expression of two proteins, METTL1 and WDR4, is abnormally elevated in ESCC and is associated with disease progression. Targeted treatment of these two proteins resulted in a reduction in the expression of specific tRNA, reducing the translation of some oncogenes, including genes associated with the mTOR signaling pathway and autophagy. This results in the overactivation of mTORC1 mediated autophagy, which induces ESCC cell apoptosis (Han et al., 2023). These findings reveal a new tRNA modified-mediated translation regulation mechanism, linking the translation mechanism to the autophagy mechanism, and suggest that METTL1 and its downstream signaling pathways may be potential targets for ESCC therapy.

### 5.6 Gastric cancer

**FIGURE 2:**
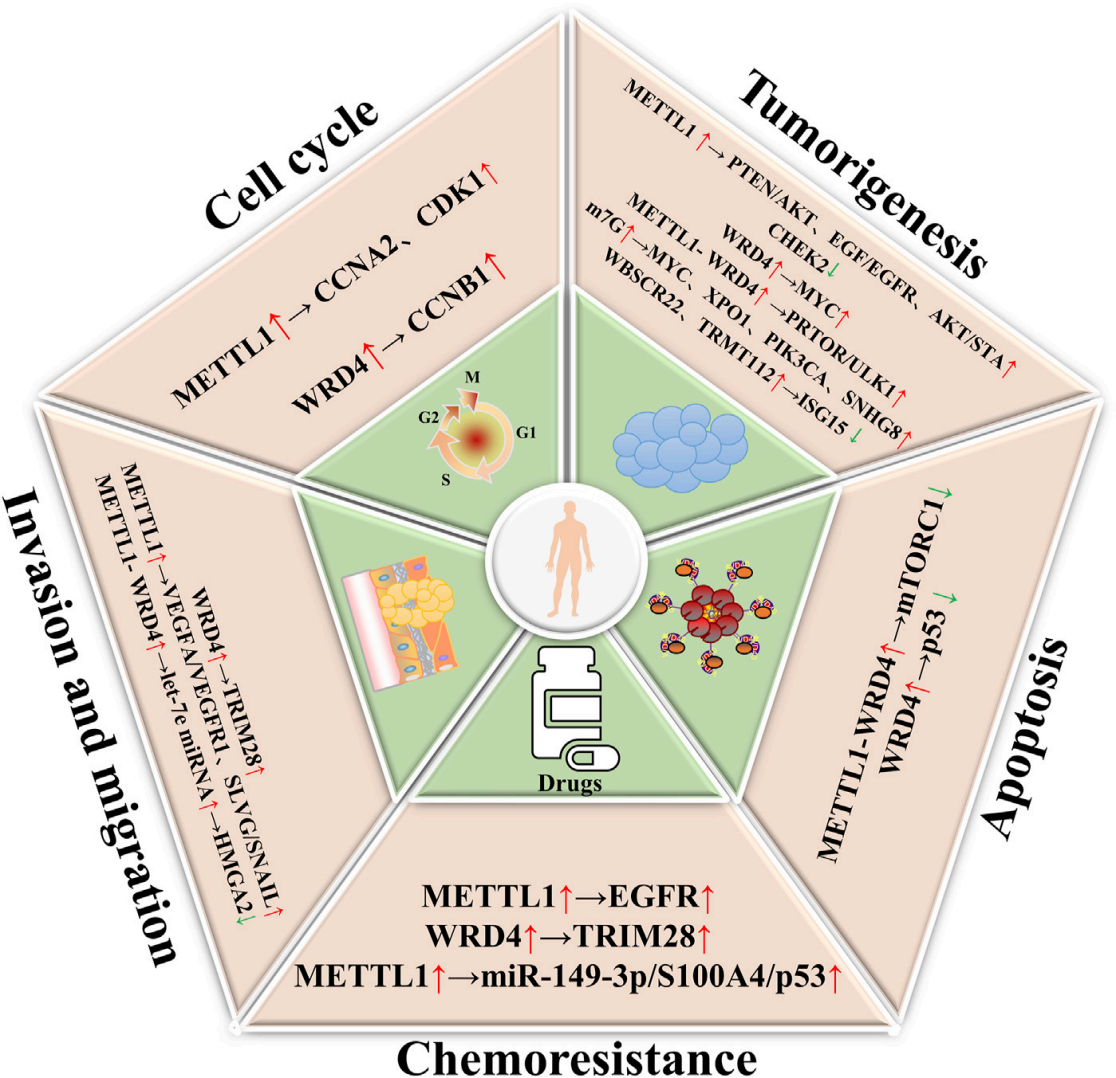
The role of m7G in digestive cancers. METTL1/WDR4, as m7G writer enzymes, can regulate cell cycle, apoptosis, invasion and migration, tumorigenesis, and chemoresistance, ultimately leading to the malignancy of digestive cancers.

Gastric cancer (GC) remains the fifth most common cancer in the world, and is the third leading cause of cancer death worldwide (Ho and Tan, 2019). RNA-binding protein (RBP) in GC prevents termination of translation, degradation of protein complexes, extension of translation, and activity of translation factors. Because RBP plays an important role in maintaining the stability of mRNA, particularly through the formation of ribonucleoprotein complexes, it has been shown in recent years to be involved in the occurrence and development of several diseases, including cancer (Dai Z. et al., 2021). METTL1 is an RBP, and the expression of METTL1 in gastric cancer is higher than that in normal tissues (Chen and Jiang, 2024). Reducing METTL1 significantly inhibited the growth of GC cells both *in vitro* and *in vivo* (Li et al., 2022b). Knockout of METTL1 leads to a decrease in cell viability, which is time-dependent and dose-dependent, and reduces the number of cell colonies. METTL1 knockout was achieved by significantly reducing levels of cell cycle markers CDK1, as well as AKT and STAT3, but had no significant effect on NF-κB p65 and SAPK/JNK. This suggests that METTL1 may play a role in the cell cycle and promote tumorigenesis by activating the AKT/STAT3/CDK1 pathway. The effect on apoptosis was not significant (Zeng et al., 2022). Therefore, METTL1 may have carcinogenic effects in GC. In addition, METTL1 or WDR4 knockouts lead to impaired cell cycle gene function, affect tRNA function, ribosome suspension and mRNA translation, and inhibit cancer development (Zeng et al., 2022). This finding suggests that the AKT/STAT3/CDK1 pathway holds promise as an innovative strategy for the future treatment of GC (Figure 2).

## 6 The role of m7G in non-tumors diseases of the digestive system

### 6.1 Chronic hepatitis B

Chronic hepatitis B (CHB) is a type of chronic hepatitis caused by the hepatitis B virus (HBV). HBV is mainly transmitted through blood, sexual contact and from mother to child, and prevalence varies in different regions. If left untreated, CHB may lead to serious complications such as cirrhosis and liver cancer (Trepo et al., 2014; Locarnini et al., 2015; Revill et al., 2019). Some studies have suggested that m7G modification may play a role in the regulation of chronic hepatitis B virus infection. The m7G related genes are closely related to immune and inflammatory responses in CHB. In order to gain a clearer understanding of m7G related differentially expressed genes (DEGs) closely related to the progression of chronic HBV infection, the relationship between m7G related DEGs and immune cells was first investigated. The results showed that some genes, such as CYFIP1, DCP2, EIF4E3 and IFIT5, were positively correlated with NK T cells and activated CD8^+^ T cells, while NUDT16 and NUDT4 were negatively correlated with most immune cells. In the course of chronic HBV infection, the infiltration of immune cells leads to repeated episodes of liver inflammation, which is one of the main reasons for promoting liver fibrosis. Therefore, CYFIP1, DCP2, EIF4E3, and IFIT5 may play an important role in CHB progression, while NUDT16 and NUDT4 may help protect CHB from further disease onset (Zhang R. et al., 2023). This finding suggests that m7G modification may be a potential target for the treatment of CHB.

### 6.2 Inflammatory bowel disease

**FIGURE 3:**
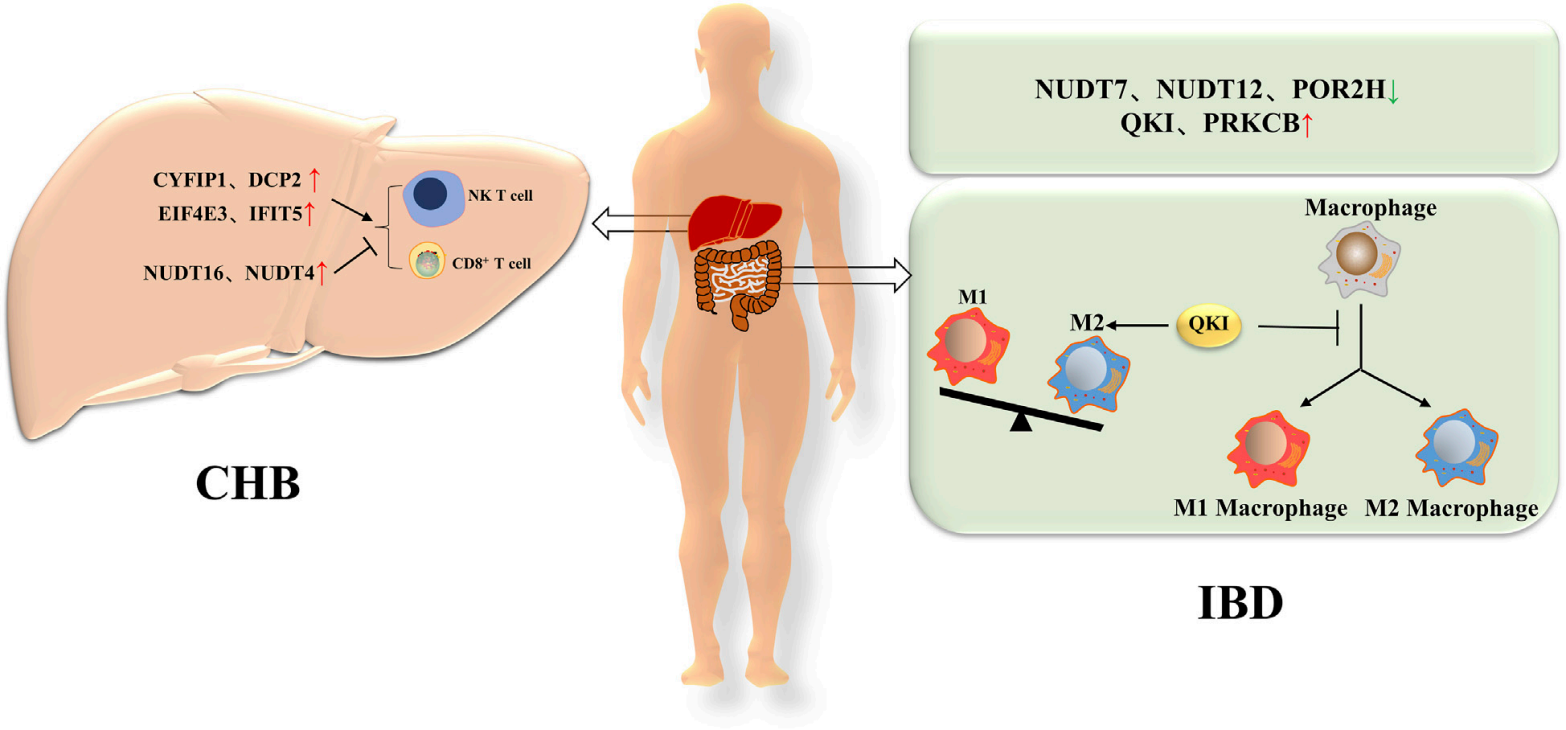
The role of m7G in non-tumors diseases of the digestive system. In CHB, m7G related genes such as CYFIP1, DCP2, EIF4E3, and IFIT5 promote the activation of NKT cells and CD8+T cells, while other m7G related genes such as NUDT16 and NUDT4 inhibit the activation of NKT cells and CD8+T cells. In IBD, there are three downregulated genes (NUDT7, NUDT12, and POLR2H) and two upregulated genes (QKI and PRKCB) associated with m7G. Among them, QKI can delay the differentiation of macrophages and lead to an increase in M2 macrophages.

Inflammatory Bowel Disease (IBD) is a group of chronic inflammatory diseases that primarily include Crohn’s disease and ulcerative colitis (UC). Both are autoimmune diseases, in which a patient’s immune system mistakenly attacks the intestinal mucosa, leading to a sustained inflammatory response. Inflammatory bowel disease is a chronic condition that requires long-term management and treatment (Zhang and Li, 2014; Flynn and Eisenstein, 2019; Bruner et al., 2023). Recent studies have shown that genes related to m7G are associated with the occurrence and development of UC. M7G is involved in various cellular activities throughout the life cycle, including cell proliferation, inflammation, and immune response. The DEGs associated with m7G are highly correlated with the level of intestinal immune cell infiltration. Our previous research indicated that there are three downregulated genes (NUDT7, NUDT12, and POLR2H) and two upregulated genes (QKI and PRKCB) in m7G related immune DEGs. In IBD, QKI delays macrophage differentiation through a negative feedback mechanism to CCAAT/enhancer binding protein (C/EBP) α. Knockout of QKI can increase the susceptibility of mice to dextran sodium sulfate (DSS) induced IBD and increase M1 macrophages. QKI-5 can reduce the translation of Keap1 mRNA, promote the activation of Nrf2, and lead to a decrease in reactive oxygen species (ROS) levels. QKI-5 also enhances antioxidant capacity by regulating the Nrf2-Keap one pathway. In the inflammatory model induced by lipopolysaccharide (LPS), QKI can penetrate nuclear factors κ B (NF- κB) pathways to regulate the polarization state of macrophages and has an impact on the severity of inflammation. QKI inhibits the expression and phosphorylation of p65, effectively inhibiting the NF-kB pathway, thereby inhibiting inflammation. Thus, QKI plays a protective role in intestinal inflammation (Wang W. et al., 2021; Yang and Yuan, 2023). (Figure 3)

## 7 The role of m7G in the tumor immune microenvironment

The tumor immune microenvironment (TIME) is a complex system that encompasses tumor cells, stromal cells, and a diverse population of immune cells, including both adaptive and innate immune cells (Kim et al., 2023). Recent studies have shown that fluctuations in the level of m7G modification can significantly affect the infiltration patterns of immune cells within the TIME. Specifically, in certain types of tumors, a high level of m7G modification has been observed to positively correlate with an increased infiltration of immune cells such as dendritic cells, B cells, and lymphocytes (Huang et al., 2022; Li D. X. et al., 2023). It is noteworthy that the TIME is not solely composed of immune cells. Tumor cells and stromal cells are also integral components (Mao et al., 2021). The latest scientific research reveals that m7G modification profoundly influences tumor cells and stromal cells in the TIME through various mechanisms. These mechanisms include the expression of immune checkpoints, the secretion of related bioactive factors, and changes in the function and communication of stromal cells (Li J. et al., 2023; Maimaiti et al., 2023).

## 8 Conclusion and prospects

In this review, we not only focus on the structure and mechanism of m7G modified writers METTL1 and WDR4, as well as reader QKI, but also on the role of m7G in the digestive system, including digestive system tumors and non-tumor diseases (Table 1). M7G holds potential application prospects as a therapeutic target for digestive system diseases. By modulating the activity of methyltransferases such as METTL1 or WDR4, the level of m7G modification can be altered, thereby regulating the expression of related genes and cellular functions, and offering new hope for the treatment of digestive diseases.

**TABLE 1:** Function of m7G in digestive diseases.

Type	Disease	m7G regulators	Expression	Pathway	Function	References
Tumor	HCC	METTL1, WDR4	Down	CCNA2, CCNB1	Cell cycle arrest	Chen et al. (2021), Dai et al. (2021a)
		METTL1, WDR4	Up	PTEN/AKT, EGF/EGFR, MYC	Promote tumorigenesis	Chen et al. (2021), Chen et al. (2022b)
		WDR4	Down	PI3K/AKT/p53	Apoptosis	Xia et al. (2021), Li et al. (2022a)
		METTL1, WDR4	Up	VEGFA/VEGFR, SLUG/SNAIL	Invasion and migration	Chen et al. (2021), Zhu et al. (2023)
		METTL1, WDR4	Up	EGFR, TRIM28	Levatinib resistance	Huang et al. (2023), Dong et al. (2024)
	CC	METTL1	Up	CHEK2	Promote tumorigenesis	Jiang et al. (2024)
		METTL1, WDR4	Up	let-7e miRNA/HMGA2	Invasion and migration	Liu et al. (2020)
		METTL1	Up	miR-149-3p/S100A4/p53	Sensitive to cisplatin	Liu et al. (2019)
	PC	SNHG8, WBSCR22, TRMT112	Up	-	Inhibiting tumor development	Luo et al. (2022), Lu et al. (2023)
	ESCC	METTL1, WDR4	Up	RPTOR/ULK1	Promote tumorigenesis	Han et al. (2022)
		METTL1, WDR4	Down	mTORC1	Apoptosis	Han et al. (2023)
	GC	METTL1	Down	CDK1	Cell cycle arrest	Zeng et al. (2022)
		METTL1	Up	AKT/STAT3	Promote tumorigenesis	Zeng et al. (2022)
Non-tumor	CHB	CYFIP1, DCP2, EIF4E3, IFIT5	Up	-	Increased immune cells	Zhang et al. (2023a)
		NUDT16, NUDT4	Down	-	Decreased immune cells	Zhang et al. (2023b)
	IBD	QKI, PRKCB	Up	Nrf 2-Keap 1, NF-κB	Antioxidation and antiinflammation	Wang et al. (2021b), Yang and Yuan (2023)

The METTL1/WDR4 complex, as a writer of m7G modification, plays an important role in the occurrence and development of many diseases. The METTL1/WDR4 complex can methylate mRNA, tRNA, and miRNA (Du et al., 2023; Ruiz-Arroyo et al., 2023) and affect translation, thus playing a role in digestive diseases. As a reader of m7G modification, QKI has a specific recognition effect in m7G modified internal mRNA. It combines with m7G modified mRNA rich in the “GA” sequence to regulate the metabolism of the target subset (Zhao et al., 2023). QKI inhibits the expression and phosphorylation of p65 and effectively inhibits the NF-kB pathway, thereby inhibiting inflammation (Wang Y. et al., 2021; Yang and Yuan, 2023). These mechanisms are closely related to the occurrence of digestive disease. Upregulation of METTL1 and WDR4 was associated with advanced tumor stage, vascular invasion status, and poor patient survival (Wang H. et al., 2023; Wang W. et al., 2024). The silencing of METTL1 and WDR4 inhibits the proliferation, migration and invasion of cancer cells, while the forced expression of METTL1 contributes to tumor progression. In the tumor immune microenvironment, m7G modification may influence the initiation and progression of tumors by regulating the infiltration and activation of immune cells. Additionally, m7G modification may also affect the immune evasion of tumor cells by regulating the expression of immune checkpoints. m7G-related DEGs was highly correlated with intestinal immune cell infiltration. This is associated with the development of chronic hepatitis B and inflammatory bowel disease (Yang and Yuan, 2023; Zhang et al., 2023).

In summary, we have determined the role of m7G in the proliferation, invasion and migration, apoptosis, and chemoresistance of digestive system tumor cells. In addition, m7G modification is related to the level of immune cell infiltration in non-tumor digestive system diseases. These results highlight the significant clinical translational value of m7G modification as a potential therapeutic target and biomarker in the treatment and diagnosis of gastrointestinal diseases, offering new insights into epigenetic research. Of course, m7G research also faces technical challenges, with limitations still existing in terms of resolution, sensitivity, and accuracy. With the continuous development of technology, it is believed that research on m7G will provide important support for the early diagnosis and personalized treatment of digestive system diseases, bringing new hope for improving the quality of life and prognosis of patients.
